# Supplementary material for: Reconciling Mining with the Conservation of Cave Biodiversity: A Quantitative Baseline to Help Establish Conservation Priorities
Source: PLoS One. 2016 Dec 20;11(12):e0168348. doi: 10.1371/journal.pone.0168348 (PMC5173368; doi:10.1371/journal.pone.0168348)
Supplement: S1 Dataset — (ZIP) [file pone.0168348.s002.zip › Taxa/Serra Norte/SN_2013/N4E/Lista N4E-22.pdf]

CAVIDADE N4E-0022

Classe	Ordem	Fam/Outros	Gên/Outros	Espécie	S-U	U-S	Zon.
Annelida	Oligochaeta			jovem		5	P/A
Arachnida	Acari	(Oribatida)		jovem			
Arachnida	Acari	(Parasitengonina)		sp.13		X	P
Arachnida	Acari	(Parasitengonina)		sp.2		X	P/A
Arachnida	Acari	(Parasitengonina)		sp.6		X	
Arachnida	Acari	(Parasitengonina)		sp.8		X	A
Arachnida	Acari	(Uropodina)		sp.3		X	A
Arachnida	Acari	Argasidae	*Ornithodoros*	sp.1	X	X	P/A
Arachnida	Acari	Eupodidae		sp.	X		
Arachnida	Acari	Holothyrida		sp.	X		
Arachnida	Acari	Prostigmata		sp.	X		
Arachnida	Acari	Trombiculidae		sp.39	X	X	P/A
Arachnida	Amblypygi	Charinidae	*Charinus*	sp.1	X	8	P/A
Arachnida	Amblypygi	Phrynidae	*Heterophrynus*	*longicornis*	X	2	E/P
Arachnida	Araneae	Araneidae	*Alpaida*	sp.1	X		
Arachnida	Araneae	Araneidae	*Micrathena*	sp.1	X		
Arachnida	Araneae	Caponiidae		jovem		X	P
Arachnida	Araneae	Corinnidae	*Creugas*	sp.1		1	A
Arachnida	Araneae	Ctenidae		jovem	1		
Arachnida	Araneae	Filistatidae		sp.1	X		
Arachnida	Araneae	Nesticidae	*Nesticus*	sp.2	X	X	A
Arachnida	Araneae	Ochyroceratidae	*Ochyrocera*	sp.	X		
Arachnida	Araneae	Ochyroceratidae	*Ochyrocera*	sp.1		X	P/A
Arachnida	Araneae	Ochyroceratidae	*Ochyrocera*	sp.3		X	A
Arachnida	Araneae	Ochyroceratidae	*Speocera*	sp.1	X	X	P/A
Arachnida	Araneae	Oonopidae	aff. *Xyccarph*	sp.1		X	P
Arachnida	Araneae	Pholcidae	*Mesabolivar*	sp.1	X	X	E/A
Arachnida	Araneae	Salticidae		sp.	X		
Arachnida	Araneae	Scytodidae	*Scytodes*	sp.	X		
Arachnida	Araneae	Segestriidae		jovem	X		
Arachnida	Araneae	Theraphosidae	aff. *Holothele*	sp.1		2	E
Arachnida	Araneae	Theridiidae		sp.	X		
Arachnida	Araneae	Theridiosomatidae	*Plato*	sp.1	X	X	E/P/A
Arachnida	Araneae	Uloboridae	*Uloborus*	sp.	X		
Arachnida	Opiliones	Escadabiidae		sp.1	X	X	P
Arachnida	Opiliones	Escadabiidae		sp.2	X	X	P/A
Arachnida	Opiliones	Stygnidae	*Protimesius*	*gracilis*	X		
Arachnida	Palpigradi	Eukoeneniidae	*Allokoenenia*	sp.1		X	A
Arachnida	Pseudoscorpiones	Bochicidae		sp.1	X	X	P
Arachnida	Pseudoscorpiones	Chernetidae	*Spelaeochernes*	sp.1	X	X	P/A
Arachnida	Pseudoscorpiones	Chernetidae		sp.2		X	
Arachnida	Pseudoscorpiones	Chthoniidae	*Pseudochthonius*	sp.1	X	X	P/A
Arachnida	Pseudoscorpiones	Chthoniidae	*Pseudochthonius*	sp.3		X	P
Chilopoda	Scolopendromorpha	Scolopocryptopidae	*Newportia*	sp.	X		
Chilopoda	Scutigeromorpha	Pselliodidae	*Sphendononema*	*guildingii*	X		
Diplopoda	Glomeridesmida	Glomeridesmidae		sp.	X		
Diplopoda	Polydesmida	Chelodesmidae		jovem		1	A
Diplopoda	Polydesmida	Pyrgodesmidae		sp.1	X	X	P/A
Diplopoda	Spirostreptida	Pseudonannolenidae	*Pseudonannolene*	jovem		17	P
Entognatha	Collembola	Isotomidae		sp.1		X	P/A
Entognatha	Collembola	Paronellidae		sp.1		X	A
Entognatha	Collembola	Paronellidae		sp.4		X	E/P
Entognatha	Collembola	Sminthuroidea		sp.2	X	X	P/A
Entognatha	Diplura	Campodeidae		sp.1	X	X	P
Insecta	Blattodea	Blattellidae		sp.1		4	P

Insecta	Blattodea	Polyphagidae		jovem		1	P
Insecta	Blattodea			jovem	X	2	P
Insecta	Coleoptera	Carabidae		sp.16	X	X	A
Insecta	Coleoptera	Dytiscidae	*Copelatus*	*cessaima*	X	X	A
Insecta	Coleoptera	Dytiscidae		sp.16		X	A
Insecta	Coleoptera	Elateridae		sp.	X		
Insecta	Coleoptera	Staphylinidae		sp.34	X	X	
Insecta	Coleoptera	Tenebrionidae		jovem	X		
Insecta	Diptera	Cecidomyiidae	Cecidomyiinae	sp.		X	P
Insecta	Diptera	Culicidae	*Culex*	sp.	X	X	P/A
Insecta	Diptera	Curtonotidae		sp.	X		
Insecta	Diptera	Dolichopodidae		sp.		X	P/A
Insecta	Diptera	Drosophilidae	*Drosophila*	sp.	X		
Insecta	Diptera	Mycetophilidae		sp.	X		
Insecta	Diptera	Phoridae	Phorinae	sp.	X	X	P
Insecta	Diptera	Psychodidae	*Breviscapus*	sp.		X	P
Insecta	Diptera	Psychodidae	*Edentomyia*	*piauiensis*	X		
Insecta	Diptera	Psychodidae	Phlebotominae	sp.		X	P/A
Insecta	Diptera	Psychodidae	*Pintomyia*	*pacae*	X		
Insecta	Diptera	Sciaridae		sp.		X	P
Insecta	Diptera	Tipulidae	Tipulinae	sp.		X	A
Insecta	Heteroptera	Cydnidae		sp.		X	A
Insecta	Heteroptera	Dipsocoroidea		sp.		X	A
Insecta	Heteroptera	Reduviidae	*Dohrnemesa*	sp.	X		
Insecta	Homoptera	Cixiidae		sp.1	X	X	A
Insecta	Hymenoptera	Bethylidae		sp.1		X	A
Insecta	Hymenoptera	Diapriidae		sp.1		X	P
Insecta	Hymenoptera	Diapriidae		sp.2		X	P/A
Insecta	Hymenoptera	Formicidae	*Camponotus*	*atriceps*	X		
Insecta	Hymenoptera	Formicidae	*Camponotus*	sp.1		X	P/A
Insecta	Hymenoptera	Formicidae	*Camponotus*	sp.2		X	P/A
Insecta	Hymenoptera	Formicidae	*Hypoponera*	sp.	X		
Insecta	Hymenoptera	Formicidae	*Hypoponera*	sp.1		X	P
Insecta	Hymenoptera	Formicidae	*Hypoponera*	sp.2		X	P
Insecta	Hymenoptera	Formicidae	*Labidus*	*coecus*	X		
Insecta	Hymenoptera	Formicidae	*Pachycondyla*	*constricta*	X		
Insecta	Hymenoptera	Formicidae	*Pachycondyla*	sp.1		X	P
Insecta	Hymenoptera	Formicidae	*Pachycondyla*	sp.2		X	E/P
Insecta	Isoptera	Termitidae	*Nasutitermes*	sp.	X	X	P/A
Insecta	Lepidoptera	Tineoidea		jovem	X		
Insecta	Lepidoptera	Tineoidea		sp.2		X	P
Insecta	Lepidoptera	Tineoidea		sp.3		X	A
Insecta	Orthoptera	Phalangopsidae	*Paraclodes*	sp.		1	E
Insecta	Orthoptera	Phalangopsidae	*Phalangopsis*	sp.	X	252	P/A
Insecta	Psocoptera	Psyllipsocidae		jovem	X	X	A
Insecta	Siphonaptera			sp.		X	A
Insecta	Thysanura	Lepismatidae		sp.	X		
Insecta	Thysanura	Ateluridae	Atelurinae	sp.	X		
Gastropoda	Pulmonata	Bulimulidae	*Naesiotus*	sp.		X	P
Gastropoda	Pulmonata	Subulinidae	*Lamellaxis*	sp.	X		
Gastropoda	Pulmonata	Subulinidae	*Obeliscus*	sp.	X		
Malacostraca	Isopoda	Armadillidae		sp.1		X	P
Malacostraca	Isopoda	Scleropactidae	*Circoniscus*	*buckupi*	X		
Nematoda				sp.1		X	P/A
Amphibia	Anura			sp.	1	2	P/A
Mammalia	Chiroptera	Phyllostomidae	*Carollia*	*perspicillata*		13	P/A
Mammalia	Chiroptera	Phyllostomidae	*Glossophaga*	*soricina*		10	P/A
Mammalia	Chiroptera	Phyllostomidae	*Lonchorrhina*	*aurita*		7	P/A

Mammalia	Chiroptera			sp.	50		P/A
Mammalia	Rodentia	Cricetidae	*Rhipidomys*	sp.		1	A
